# Serum and Striatal Redox and Metabolic Responses to Progesterone Treatment in Rats with Common Carotid Ligation

**DOI:** 10.3390/antiox15050610

**Published:** 2026-05-12

**Authors:** Ivana Guševac Stojanović, Ana Todorović, Filip Veljković, Katarina Bobić, Jelena Martinović, Snežana Pejić, Suzana Veličković, Zoran Stojanović, Dunja Drakulić

**Affiliations:** 1 Department of Molecular Biology and Endocrinology, VINČA Institute of Nuclear Sciences–National Institute of the Republic of Serbia, University of Belgrade, 11001 Belgrade, Serbia; igusevac@vin.bg.ac.rs (I.G.S.); anato@vin.bg.ac.rs (A.T.); katarina.bobic@vin.bg.ac.rs (K.B.); jelenazlatkovic@vin.bg.ac.rs (J.M.); snezana@vin.bg.ac.rs (S.P.); 2 Department of Physical Chemistry, VINČA Institute of Nuclear Sciences–National Institute of the Republic of Serbia, University of Belgrade, 11001 Belgrade, Serbia; filipveljkovic@vin.bg.ac.rs (F.V.); vsuzana@vin.bg.ac.rs (S.V.); 3 Group for Biomaterials and Biomedical Applications, Institute of Technical Sciences of SASA, 11000 Belgrade, Serbia; zoran.stojanovic@itn.sanu.ac.rs

**Keywords:** chronic cerebral hypoperfusion (CCH), progesterone, serum, striatum, oxidative stress, lipid metabolism, adenine nucleotide turnover, crude synaptosomal fraction

## Abstract

Cerebrovascular and neurodegenerative diseases are often linked to dysregulated cerebral blood flow, which results in oxidative stress and alterations in energy metabolism. Targeting the underlying initiators and exacerbating factors could offer protective benefits. Among the proposed therapeutic agents, the steroid hormone progesterone (P4) has shown considerable potential. This study evaluates the protective effects of P4 (1.7 mg/kg, administered subcutaneously once daily for a week) in a rat model of chronic cerebral hypoperfusion (CCH), provoked by the permanent bilateral ligation of the common carotid arteries. Redox and metabolic imbalances, specifically lipid and adenine nucleotide metabolism, were examined in serum and striatal crude synaptosomal fractions. Additionally, sensorimotor functions were assessed using non-invasive neurological tests. Biochemical analyses showed that P4 in CCH conditions contributed to the normalization of redox and metabolic homeostasis in both the serum and striatum. In the serum, this was accompanied by increased adenine nucleotide turnover, likely favoring protective adenosine signaling. In parallel, P4 alleviated the striatal oxidative burden while augmenting antioxidant response and promoting nucleotide catabolism. Our findings demonstrate that P4-mediated protection is accomplished through coordinated biochemical serum–striatum responses, linking systemic and synaptic metabolic regulation with improved sensorimotor function and recovery from CCH-induced deficits.

## 1. Introduction

The number of individuals with cerebrovascular and neurodegenerative diseases is rising rapidly as the world population ages [1,2]. The usual features of these age-related conditions are loss of memory and higher cortical functions; physical disabilities; changes in mood, personality, and behavior; and finally, death. Their onset and progression are associated with blood flow dysregulation and chronic cerebral hypoperfusion (CCH) [3,4]. CCH-provoked changes occur in selective vulnerable brain regions, like the cortex, hippocampus and striatum, due to inflammation and compromised oxidative, lipid and adenosine 5′-triphosphate (ATP) metabolism [4,5,6]. Specifically, localized cerebral limited perfusion and hypoxic milieus caused by insufficient blood flow contribute to the overproduction and release of highly unstable free radicals and damage-associated molecular patterns, including membrane phospholipids (e.g., lysophosphatidylcholine, LPC) and components of purinergic signaling. These species damage surrounding biomolecules, modulate the actions of antioxidative defense components like superoxide dismutase (SOD) and catalase (CAT), additionally exacerbate the generation of free radicals, promote a pro-inflammatory microenvironment [7,8] and potentially induce cell death. This is further reflected in altered antioxidant responses within the central nervous system during neurodegeneration [9], pointing out oxidative stress as an important contributor to secondary tissue damage. Furthermore, CCH results in the release of purine nucleotides, like ATP/adenosine 5′-di/monophosphates (ADP/AMP) and adenosine, important cellular metabolites with both instant and extended time-course trophic outcomes [7]. ATP is, for one, recognized as a pro-inflammatory agent since it directs the recruitment of immune cells towards damaged areas and promotes leukocyte–endothelial adhesion [10] and the secretion of pro-inflammatory cytokines from various immune cells [11,12]. Released cytokines provoke the activation of T-lymphocytes and macrophages, creating an inflammatory environment. The amount of ATP in the extracellular space is regulated by members of the ectonucleoside triphosphate diphosphohydrolase family, including CD39, that convert ATP to either ADP or directly to AMP. Newly formed AMP is further hydrolyzed by CD73 (ecto-5′-nucleotidase, eN) to adenosine, a modulatory, antioxidative, anti-inflammatory, immunosuppressive and protective agent [13,14]. Both CD39 and CD73 function as surface-anchored enzymes in various cellular populations, though they also circulate within physiological fluids as soluble forms [15,16,17,18,19].

Effective pharmacological compounds that would completely delay or prevent CCH-induced abnormal changes in the production and elimination of free radicals and energy metabolites and/or increase cellular defense mechanisms are still unavailable. However, the literature provides strong evidence about the multifaceted protective actions of the steroid hormone progesterone (P4) through both systemic and local mechanisms [20,21,22,23]. These include a higher survival rate, the promotion of functional recovery, the maintenance or restoration of the blood–brain barrier, and a reduction in cerebral edema and the volume size of lesions. Such P4 outcomes are most likely mediated by improvements in local energy metabolism, the regulation of microglial activation, and apoptotic and inflammatory responses, as well as other signaling pathways [24,25,26,27,28,29,30,31,32]. Despite these findings, the potential coordination between systemic metabolic responses and local striatal changes following P4 treatment in CCH remains poorly defined, although understanding this association is essential for evaluating its therapeutic efficacy. Therefore, we examined the neuroprotective capacity of P4 in rats that underwent the permanent ligation of both common carotid arteries by analyzing striatal and serum redox status, lipid metabolism, and adenine nucleotide turnover, alongside neurological performance.

## 2. Materials and Methods

### 2.1. Chemicals

We used reagents and chemicals of analytical grade and the maximum commercially available purity, obtained from either LGC Promochem GmbH (Wesel, Germany) or Sigma-Aldrich Co. (St. Louis, MO, USA).

### 2.2. Animals

For the purpose of this study, we used adult male Wistar albino rats (300–350 g) provided by our in-house, pathogen-free colony in the vivarium at VINČA Institute of Nuclear Sciences, National Institute of the Republic of Serbia, University of Belgrade, Belgrade, Republic of Serbia. Animals were maintained in a controlled, standardized environment. We followed the strict welfare standards of the European Community Council (Directives 86/609/EEC and 2010/63/EU), while each experimental procedure was carefully planned to align with the 3R principles, ensuring the highest level of care while using the minimum number of animals necessary. The experimental protocol approval numbers are 02/11 (issued 20 February 2014), 323-07-04253/2016-05 (issued 16 May 2016), and 04/2023 (issued 7 August 2023).

### 2.3. Surgical Procedure and Treatment

Anesthesia was induced with a single intraperitoneal dose of chloral hydrate (400 mg/kg) prior to surgeries. Rats’ necks were incised, and both common carotid arteries were carefully separated from the carotid sheath and cervical sympathetic and vagus nerves. The animals were then randomly allocated to three groups (*n* = 6 per group): (I) controls that underwent surgery without occlusion of carotid arteries followed by the treatment with the vehicle (commercial flax oil, dose 1 mL/kg/day, Sham+V); (II) rats with permanently ligated carotid arteries treated with the vehicle in dose 1 mL/kg/day (2VO+V); (III) rats with permanently ligated carotid arteries that received P4 dissolved in the vehicle in dose 1.7 mg/kg/day (2VO+P4).

The vehicle or P4 treatments, administered subcutaneously, lasted for 7 consecutive days. The number of animals, dose and treatment regimen were chosen on the basis of our previous studies demonstrating neuroprotection effects in other brain regions and in line with the principles of reduction in animal experimentation [24,29].

### 2.4. Neurological Testing

A battery of non-invasive neurological tests was conducted by the researcher blinded to the experimental setup who assessed the potential sensorimotor deficits before the surgical procedures and treatments and on the day of sacrifice [33]. The scoring ranged from 0 to 2 points per examined parameter, as follows: consciousness, 0 or 1; respiration, 0 or 1; pacing/circling, 0 or 1; behavior on horizontal platform, 0, 1 or 2; behavior on an inclined platform, 0, 1 or 2; grip strength, 0, 1 or 2; visual forepaw reaching, 0 or 1. The total score of sensorimotor deficits amounts to the sum of average points of all seven tested parameters per group/day, with the final score between 0 and 10 per animal.

### 2.5. Sample Collection and Preparation

Following decapitation (Harvard Apparatus, Holliston, MA, USA) 4 h after the last treatment, isolated striatal tissues were immediately snap-frozen and stored at −80 °C for the subsequent obtaining of crude synaptosomal fractions [34,35]. In parallel, blood was collected for serum preparation (30 min clotting period at room temperature, followed by 15 min of centrifugation at 3000× *g*). The resulting supernatant was then isolated and maintained at −80 °C until analyses.

### 2.6. Total Protein Determination

Protein concentrations were quantified using the modified method of Lowry [36], with bovine serum albumin serving as the reference standard for calibration.

### 2.7. Estimation of Redox Parameters

The serum and striatal levels of prooxidant/antioxidant balance (PAB), advanced oxidative protein products (AOPP), and the end products of lipid peroxidation (LPO) were assayed in duplicate on 96-well plates using a Tecan Sunrise microplate reader (Tecan Group Ltd., Männedorf, Switzerland). With modifications to the original method described by Alamdari et al. [37], PAB levels were measured at 450 nm via a TMB-based reaction, while the results expressed in HK units represent the percentage of hydrogen peroxide (H_2_O_2_) in the standard mixture. The levels of AOPP, measured at 340 nm using potassium iodide, phosphate-buffered saline (pH 7.4) and acetic acid, were expressed as µmol/L chloramine-T equivalents based on the method of Witko-Sarsat et al. 1996 [38], with minor modifications. An adapted method from Gérard-Monnier et al. 1998 [39], was used for the estimation of LPO in reaction mixtures containing samples and working solutions with methanesulfonic acid, at 580 nm, with the results expressed in µM. Additionally, SOD and CAT activities were analyzed using an S-40 Boeco spectrophotometer (Hamburg, Germany). Specifically, SOD activities were measured at 480 nm based on the inhibition of adrenaline autoxidation [40], where one unit of SOD activity was defined as the amount of enzyme required to inhibit 50% of the reaction. CAT activity was assayed at 240 nm by monitoring the decomposition of H_2_O_2_ [41], where one unit represented 90% substrate destruction per min. Enzymatic activities were expressed as U/mg of tissue protein or U/mL for serum.

### 2.8. Assays for Nucleotide Hydrolysis

The hydrolysis of ATP and ADP was conducted in a 200 µL reaction mixture (112.5 mmol/L Tris-HCl, pH 8.0; 0.5 mmol/L EDTA; 5 mmol/L MgCl_2_) containing 0.5 mg serum proteins and 0.5 mmol/L substrate (ATP/ADP). AMP hydrolysis was assayed with 0.5 mg protein and 0.5 mmol/L AMP separately under modified conditions (112.5 mmol/L Tris-HCl (pH 8.0) and 10 mmol/L MgCl_2_). All reactions (40 min, 37 °C) were stopped with 3 mol/L perchloric acid (PCA) at 4 °C. To account for non-enzymatic Pi release, blanks were prepared by adding serum proteins post-acidification. Following centrifugation (5000× *g*, 5 min, 4 °C), supernatant Pi content was determined at 650 nm using malachite green [42].

The striatal ATP and ADP hydrolysis rates were estimated in 200 µL of the reaction mixture (50 mmol/L Tris-HCl, pH 7.4; 5 mmol/L MgCl_2_) with 10 µg of protein, while for AMP hydrolysis, the mixture contained 100 mmol/L Tris-HCl (pH 7.4), 10 mmol/L MgCl_2_ and 20 µg of protein. After 10 min preincubation at 37 °C, the substrate (1 mmol/L) was added. Incubation lasted 15 min for ATP/ADP and 30 min for AMP. Reactions were stopped with 3 M PCA on ice. Following centrifugation, Pi release was determined at 650 nm using malachite green [43].

### 2.9. Lipids Analysis by MALDI-TOF Mass Spectrometry

Total lipids from serum and striatal crude synaptosomal fractions (50 μL) were extracted following the Folch protocol [44], with a solvent ratio of 2:1:0.4 (*v*/*v*/*v*) to ensure distinct phase separation and reliable recovery within the organic layer. For the determination of the phosphatidylcholine (PC)-to-LPC ratio, 1 µL of the resulting organic extract combined with matrix solution (2,5-dihydroxybenzoic acid and 0.5 M 1,1,3,3-tetramethoxypropane in 70:30 methanol/water with 0.1% trifluoroacetic acid) was deposited onto a stainless steel target plate and air-dried. Mass spectra were recorded using a MALDI-TOF spectrometer (Voyager Biospectrometry DE Pro, PerSeptive Biosystems, Framingham, MA, USA) under in-house established conditions (positive reflector mode, delayed extraction with a 337 nm nitrogen laser, 150 shots per spectrum, 3600 a.u., and an acceleration voltage of 20 kV).

### 2.10. Data Analysis

All data are expressed as a percentage of the mean of the values in Sham+V ± SEM. Statistical analyses were performed using GraphPad Prism 8.02 software (GraphPad Software, Boston, MA, USA). The analysis of obtained data with a Gaussian distribution was done by a one-way analysis of variance (ANOVA) test followed by Tukey’s post hoc test, while the data without normal distribution were analyzed by a Kruskal–Wallis test followed by Dunn’s multiple comparison test. *p* < 0.05 was considered significant.

## 3. Results

Pre-operative neurological assessment confirmed that all animals exhibited normal sensorimotor function (Table 1). Prior to sacrifice, both 2VO groups showed a decline in sensorimotor functions compared to the Sham+V group (*p* < 0.001 for 2VO+V and *p* < 0.05 for 2VO+P4) (Table 1). As presented in Table 1, rats in 2VO+P4 showed a less pronounced sensorimotor deficit than those in the 2VO+V group (*p* < 0.001).

**Figure 1:**
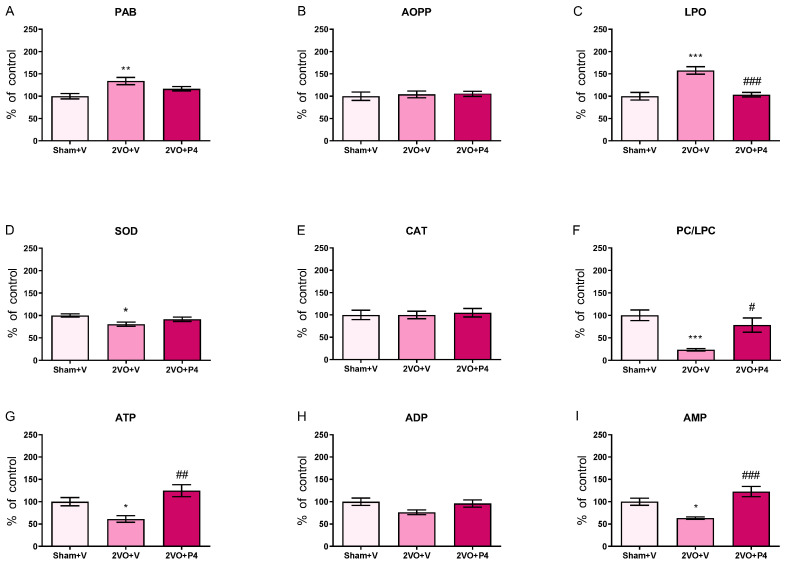
Levels of oxidative stress indicators and adenine nucleotide hydrolysis rates in serum of sham-operated animals treated with vehicle (Sham+V) and rats subjected to permanent ligation of carotid arteries treated with either vehicle (2VO+V) or progesterone (2VO+P4). Levels of prooxidant/antioxidant balance (PAB) (**A**); advanced oxidative protein products (AOPP) (**B**); end products of lipid peroxidation (LPO) (**C**); activities of superoxide dismutase (SOD) (**D**) and catalase (CAT) (**E**); signal-to-noise ratios of most abundant phosphatidylcholines (PC) and lysophosphatidylcholines (LPC) (**F**); adenosine nucleotide (ATP, ADP and AMP) hydrolysis rates (**G**–**I**). Data are presented as mean ± SEM (*n* = 6 per group), with Sham+V values set as 100%. Statistical analysis was performed using ANOVA followed by Tukey’s post hoc test (* *p* < 0.05, ** *p* < 0.01, *** *p* < 0.001 for comparison between Sham+V and either 2VO+V or 2VO+P4; ^#^ *p* < 0.05, ^##^ *p* < 0.01, ^###^ *p* < 0.001 for comparison between 2VO+V and 2VO+P4).

Compared to the Sham+V group, 2VO+V animals exhibited significantly elevated serum PAB and LPO levels (*p* < 0.01 and *p* < 0.001, respectively) (Figure 1A,C). These changes were accompanied by decreased SOD activity and a reduced signal-to-noise (S/N) ratio of PC/LPC along with lower ATP and AMP hydrolysis rates (*p* < 0.001 for PC/LPC; *p* < 0.05 for SOD, ATP and AMP) (Figure 1D,F,G,I). In the 2VO group, treatment with P4, with respect to the vehicle, attenuated detected alterations, as demonstrated by reduced LPO levels (*p* < 0.001) (Figure 1C) and increased PC/LPC together with ATP and AMP hydrolysis rates (*p* < 0.001 for AMP; *p* < 0.01 for ATP; *p* < 0.05 for PC/LPC) (Figure 1F–H). As illustrated in Figure 1, the Sham+V and 2VO+P4 groups exhibited similar values for all analyzed parameters, indicating a return toward control levels in 2VO+P4 rats.

**Figure 2:**
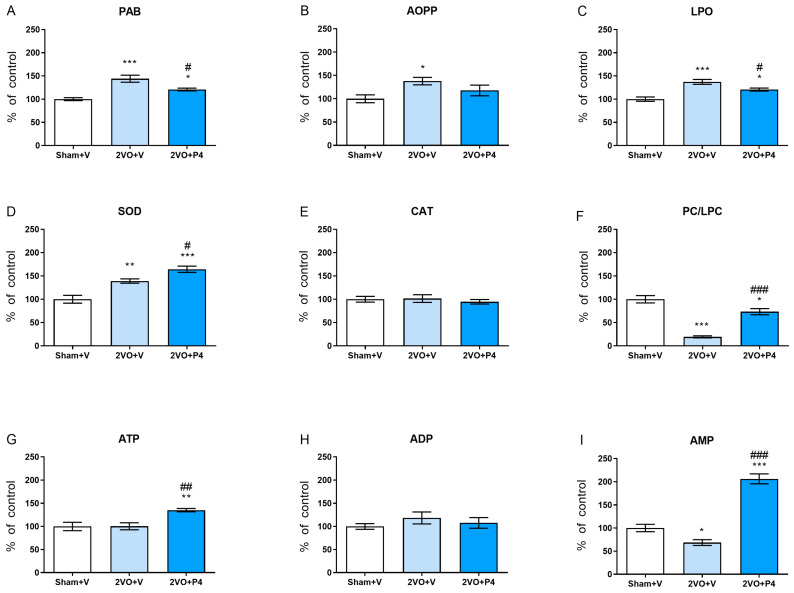
Levels of oxidative stress indicators and adenine nucleotide hydrolysis rates in striatum of sham-operated animals treated with vehicle (Sham+V) and rats subjected to permanent ligation of carotid arteries treated with either vehicle (2VO+V) or progesterone (2VO+P4). Levels of prooxidant/antioxidant balance (PAB) (**A**); advanced oxidative protein products (AOPP) (**B**); end products of lipid peroxidation (LPO) (**C**); activities of superoxide dismutase (SOD) (**D**) and catalase (CAT) (**E**); signal-to-noise ratios of most abundant phosphatidylcholines (PC) and lysophosphatidylcholines (LPC) (**F**); adenosine nucleotide (ATP, ADP and AMP) hydrolysis rates (**G**–**I**). Data are presented as mean ± SEM (*n* = 6 per group), with Sham+V values set as 100%. Statistical analysis was performed using ANOVA followed by Tukey’s post hoc test (* *p* < 0.05, ** *p* < 0.01, *** *p* < 0.001 for comparison between Sham+V and either 2VO+V or 2VO+P4; ^#^ *p* < 0.05, ^##^ *p* < 0.01, ^###^ *p* < 0.001 for comparison between 2VO+V and 2VO+P4).

In the striatum, 2VO+V rats had significantly augmented levels of PAB, AOPPs and LPO along with increased SOD activity compared to controls (*p* < 0.001 for PAB and LPO; *p* < 0.01 for SOD; *p* < 0.05 for AOPP) (Figure 2A–D). In parallel, PC/LPC and AMP hydrolysis were decreased (*p* < 0.001 and *p* < 0.05, respectively) (Figure 2I). In ligated animals, P4 treatment, relative to the vehicle, mitigated these changes, as reflected by reduced PAB and LPO levels (*p* < 0.05) (Figure 2A,C) and increased SOD activity, as well as PC/LPC, ATP and AMP hydrolysis rates (*p* < 0.001 for AMP and PC/LPC; *p* < 0.01 for ATP; *p* < 0.05 for SOD) (Figure 2D,G,I). However, with respect to Sham+V, in 2VO+P4 animals, striatal PAB and LPO contents were still elevated, whereas higher SOD activity, along with ATP and AMP hydrolysis rates (*p* < 0.001 for SOD and AMP; *p* < 0.01 for ATP; *p* < 0.05 for PAB and LPO) (Figure 2A,C,D,G,I) and decreased PC/LPC (*p* < 0.05) (Figure 2F), suggests a compensatory response, indicating only the partial normalization of striatal biochemical parameters.

## 4. Discussion

Throughout the years, numerous experimental models, including those of thromboembolism, multiple infarcts, and vessel occlusion, have been exploited to investigate the pathophysiological mechanisms underlying vascular cognitive impairment (VCI) and vascular dementia (VAD), as well as the pharmacological effects of potential drugs [45]. By counteracting the detrimental effects of oxidative stress, and various imbalances in neuronal metabolism, P4 has emerged as effective when blood flow in the specific brain regions is decreased and/or dysregulated [24,26,28,29,30,31]. Our study further elucidates this understanding by demonstrating that the protective effect of P4 against CCH suggests coordination between systemic (whole-body) and local (CNS-specific) actions, a mechanism that underlies the observed improvement in neurological deficit.

Although not considered a classical antioxidant, P4 has been shown to modulate energy metabolism, suppress excessive free radical production, upregulate cellular defenses, and thereby mitigate oxidative damage [24,46,47,48,49,50]. In line with its previously demonstrated effects, in our CCH model, P4 treatment substantially restored 2VO-induced disruptions in serum overall redox balance, lipid peroxidation, and SOD activity. This effect may, at least in part, be associated with the P4-induced modulation of mitochondrial metabolism, which could enhance respiratory efficiency and limit excessive electron leakage and free radical formation. Together, these changes may help restrain the propagation of oxidative damage, contributing to improved metabolic homeostasis rather than reflecting a direct free radical scavenging action of P4 [23,47,49]. The observed ability of P4 to reduce oxidative burden and to reinforce endogenous antioxidant capacity may indicate that this hormone contributes to a more favorable redox environment, potentially enhancing cellular resilience under CCH. Through binding to nuclear progesterone receptors (PRs), P4 has been reported to stimulate the transcription of key antioxidant genes like SOD, glutathione peroxidase (GPx), CAT, and glutathione reductase, thereby supporting oxidative stress tolerance via a receptor-dependent genomic pathway [51]. Furthermore, it upregulates the expression of complex IV and SOD2, which consequently decreases mitochondrial ROS production and increases respiratory activity [49]. While our data point to the involvement of these metabolic pathways, direct measurements of mitochondrial respiration and efflux are still needed to confirm our hypothesis. Concomitantly, P4 treatment reversed the CCH-induced decrease in the PC-to-LPC ratio, indicating a stabilizing impact on phospholipid metabolism and composition. This phenomenon may be associated with changes in the activity of phospholipase A2 (PLA2), an enzyme involved in PC turnover to LPCs and free fatty acids, which are bioactive lipids linked to lipid peroxidation, membrane disturbances, and pro-inflammatory signaling [52,53]. The literature emphasizes that P4 may modulate PLA2 activity through genomic and non-genomic mechanisms, including the induction of endogenous inhibitors such as uteroglobin, or the transcriptional regulation of phospholipase-related genes [54,55,56]. Besides its protective action on the investigated parameters of oxidative stress and phospholipid metabolism, P4 also affected CCH-provoked disturbances in serum nucleotide levels, influencing nucleotide-degrading pathways and energy homeostasis, an effect that has been scarcely investigated in the current animal model system. At the same time, the observed P4-induced augmentation in ATP and AMP hydrolysis rates points to a shift in extracellular nucleotide metabolism, suggesting changes in purinergic signaling. This likely reflects the enhanced conversion of ATP to adenosine, which may help limit excessive extracellular ATP accumulation while increasing adenosine availability. These changes are generally associated with reduced oxidative stress and excitotoxicity, as well as the modulation of immune responses and anti-inflammatory signaling [15,57,58,59]. Enzymes, including CD39 and CD73, which are present both on circulating lymphocytes and in soluble forms in body fluids, are known to regulate these processes [15,57]. Collectively, our findings suggest the coordinated modulation of lipid and purinergic pathways by P4 under CCH conditions. However, as neither mitochondrial respiratory activity or metabolic efflux nor PLA2 or CD39/CD73 expression or activities were directly assessed in the present study, these interpretations should be considered with caution.

Upon its systemic regulatory actions observed in serum, P4 concurrently attenuated oxidative and metabolic alterations in the striatum, a subcortical area critical for motor control and cognitive integration that is highly susceptible to such imbalances in ischemic state due to its unique vascular supply and high metabolic demand. This was likely accomplished through an enhancement in endogenous antioxidant defenses and purine catabolism, along with the limited release of pro-inflammatory mediators. The observed moderate reduction in overall oxidative burden, accompanied by alleviated lipid peroxidation and protein oxidation and the incomplete recovery of phospholipid turnover, suggests the partial normalization of striatal biochemical parameters and indicates that P4 may protect not only lipids but also proteins. By acting as a stabilizer of membrane phospholipid composition and lipid homeostasis, P4 may also modulate enzymatic activity and intracellular signaling, thereby preserving neuronal integrity under CCH conditions. The P4-induced stimulation of the endogenous enzymatic defense system is reflected in increased striatal SOD activity. While this enzyme is responsible for the clearance of detrimental species via the conversion of superoxide to H_2_O_2_, such an increase is not necessarily a purely protective outcome. Instead, it likely represents a compensatory and adaptive response to ongoing oxidative stress, showing that redox homeostasis has not yet been restored. Regardless of the underlying cause of SOD activation, its functional significance depends on the coordinated action of downstream antioxidant enzymes responsible for H_2_O_2_ detoxification, primarily CAT and GPx [60]. The literature emphasizes that CAT, mainly localized in peroxisomes, predominates at high H_2_O_2_ concentrations, whereas GPx, present in the cytosol and mitochondria, plays a key role at lower H_2_O_2_ levels [61,62]. The similar CAT activity observed in all experimental groups in our study suggests that other antioxidant systems may be responsible for H_2_O_2_ detoxification under these conditions, including GPx. Nonetheless, as neither GPx activity nor H_2_O_2_ levels were directly measured, their involvement remains to be experimentally confirmed in future studies. Moreover, another pivotal mechanism underlying P4’s beneficial effect is its capacity to regulate purinergic homeostasis through a significant enhancement in ATP and AMP hydrolysis, which may reflect the upregulation of CD39 and CD73 within the crude synaptosomal fraction. They potentially limit the accumulation of extracellular ATP, a well-known prooxidant and pro-inflammatory signaling molecule, thereby preventing ATP-mediated neurotoxicity while simultaneously promoting the formation of neuroprotective adenosine and contributing to the modulation of the neuro-inflammatory environment [15,57]. The observed protection of striatal protein and lipid integrity, together with enhanced purine nucleotide turnover, likely establishes the biochemical milieu necessary for the improved sensorimotor performance observed in the P4-treated rats. Although these functional improvements are relatively small in magnitude, their consistency suggests that they may reflect a biologically relevant response, representing the functional outcome of both systemic and local metabolic preservation. In the context of CCH, these subtle enhancements serve as indicators of the integrated resilience provided by P4 treatment across multiple levels.

Despite the novel insights our study provides into the multi-level redox and metabolic protection exerted by P4, certain limitations of these findings should be considered. First, regarding the experimental subjects, we used exclusively adult male rats to avoid the confounding effects of fluctuating endogenous hormones. Although this ensures a more stable experimental environment, we acknowledge that it limits the generalizability of our findings, especially given the hormonal nature of the P4 treatment; therefore, the inclusion of female subjects in future studies is essential to address potential sex-specific responses. Second, we utilized a single, targeted time point following CCH induction, as this interval was strategically selected based on our previous longitudinal data (covering 3, 7, and 90 days post-surgery in the hippocampus and prefrontal cortex) identifying it as the peak of metabolic and neuropharmacological alterations [34,35]. Nevertheless, the lack of a longitudinal perspective restricts our understanding of the temporal dynamics of P4’s effects. Similarly, the dose, regimen, and duration of treatment were chosen based on the protective potential of P4 shown in other brain regions [24,29]. Regarding the mechanistic scope, the involvement of CD39/CD73, mitochondrial metabolism, and PLA2 activity was inferred from nucleotide turnover and lipid profiles rather than direct enzymatic or expression assays. While the applied biochemical approach provides high specificity at the subcellular level, it does not offer direct mechanistic insight into the intracellular signaling pathways underlying the observed effects; consequently, these pathways should be viewed as plausible hypotheses rather than definitive conclusions. Furthermore, the absence of GPx activity assessment prevents a more comprehensive mapping of the enzymatic antioxidant response. Finally, in accordance with the 3R principles, the sample size was kept to the minimum required to achieve sufficient statistical power for our biochemical and functional analyses. This ethical constraint limited the number of animals available for histological and immunohistochemical investigations, thereby restricting our ability to directly visualize structural changes within the striatum. Consequently, while our biochemical and behavioral data strongly indicate a protective effect, they should be interpreted as evidence of metabolic and redox preservation that requires further morphological validation to definitively establish structural neuroprotection. Notwithstanding the listed limitations, our findings present a systemic–local perspective on P4’s therapeutic potential, providing a solid basis for future studies to further investigate the underlying molecular mechanisms and structural protection.

## 5. Conclusions

The current research reveals that P4 promotes coordinated systemic and local adaptive responses under ischemic conditions. This dual-site P4 action involves the attenuation of oxidative stress and an enhancement in antioxidative defense and purine nucleotide turnover, along with the regulation of phospholipid homeostasis in both the serum and striatum, which may contribute to the observed sensorimotor recovery. Although the precise intracellular signaling pathways and receptor-mediated genomic interactions underlying the multi-level protective effects of P4 remain to be fully elucidated, the presented findings provide a strong rationale for future studies aimed at validating its therapeutic potential in cerebrovascular insufficiency associated with the onset and progression of VCI and VAD.

## Figures and Tables

**Table 1:** Sensorimotor function of sham-operated animals treated with vehicle (Sham+V) and rats subjected to permanent ligation of carotid arteries treated with either vehicle (2VO+V) or progesterone (2VO+P4), evaluated on day 0 and day 7. Neurological score ranged from 0 to 10 (arbitrary units). Data are presented as mean ± SEM (*n* = 6 per group). Statistical analysis was performed using ANOVA followed by Tukey’s post hoc test (* *p* < 0.05, *** *p* < 0.001 for comparison between Sham+V and either 2VO+V or 2VO+P4, ^###^ *p* < 0.001 for comparison between 2VO+V and 2VO+P4).

Group	Sham+V	2VO+V	2VO+P4
Day 0	10.00 ± 0.00	10.00 ± 0.00	10.00 ± 0.00
Day 7	9.17 ± 0.31	5.83 ± 0.31 ***	7.83 ± 0.31 *^###^

## Data Availability

Data are contained within this article.
